# Efficacy and safety of pembrolizumab/carrelizumab, alone or in combination with chemotherapy in treatment of patients with non-small cell lung cancer: A protocol for evidence-based systematic review and Bayesian network meta-analysis

**DOI:** 10.1097/MD.0000000000026672

**Published:** 2021-07-23

**Authors:** Xiaofei Zhang, Hui Qian, Xiangkun Qu, Yalin Jiang

**Affiliations:** Department of Respiratory and Critical Care Medicine, Bozhou City People's Hospital, Bozhou Hospital affiliated to Anhui University of Technology, Anhui, China.

**Keywords:** carrelizumab, meta-analysis, non-small cell lung cancer, pembrolizumab, protocol

## Abstract

**Background:**

To date, there have been no reported trials that directly compare pembrolizumab/carrelizumab monotherapy versus pembrolizumab/carrelizumab and chemotherapy in the first-line treatment setting of advanced/metastatic non-small cell lung cancer (NSCLC). We performed a Bayesian network meta-analysis of randomized controlled trials (RCTs) to compare the efficacy and safety of pembrolizumab/carrelizumab versus pembrolizumab/carrelizumab and chemotherapy in previously treated patients with NSCLC.

**Methods:**

The following search terms would be used in PUBMED, Scopus, EMBASE, and Cochrane Library databases on July 20, 2021, as the search algorithm: (pembrolizumab) OR (carrelizumab) OR (programmed death-1) AND (non-small cell lung cancer) OR (NSCLC). All RCTs that reported the outcomes of pembrolizumab/carrelizumab with or without chemotherapy compared with those of pembrolizumab/carrelizumab alone for patients with NSCLC were considered eligible for inclusion in this meta-analysis. The primary outcomes of interest were overall survival, progression-free survival, objective response rate based on the Response Evaluation Criteria in Solid Tumors for complete and partial responses, and treatment-related adverse events including immune-related adverse events. Secondary outcomes included overall survival, progression-free survival, objective response rate, and treatment-related adverse events for the FDA-approved doses.

**Conclusions:**

The results of our review will be reported strictly following the PRISMA criteria and the review will add to the existing literature by showing compelling evidence and improved guidance in clinic settings.

**Ethical approval:**

As this study is on the basis of published or registered previous studies, ethical approval and informed consent of patients are not required.

## 1 Introduction

Lung cancer remains the leading cause of cancer death worldwide. Globally, there were an estimated 1.83 million new lung cancer cases and 1.59 million related deaths in 2012. In the United States, there will be an estimated 234,030 new cases of lung cancer in 2018, including 154,050 deaths from the disease.^[1,2]^ Non-small cell lung cancer (NSCLC) accounts for 80% to 90% of primary lung malignancies.^[3]^ NSCLC is most often diagnosed at the metastatic stage, with a 5-year survival rate of between 0% and 5% using traditional chemotherapy-based strategies. Patients with advanced/metastatic NSCLC have a poor prognosis because of limited surgical options and chemotherapy response rates, whether first-line cytotoxic or platinum-containing dual drug regimens or second-line docetaxel, of only 15% to 30%.^[4]^

Programmed death-1 is a protein expressed on activated T cells and pre-B cells as well as tumor cells, including NSCLC. Inhibition of the programmed death-1 pathway has been shown to restore T cell activity against tumor cells. Immunotherapies involving monoclonal antibodies targeting programmed death-1 (carrezumab and pembromezumab) offer an alternative for patients with NSCLC in second-line therapy.^[5–7]^ Several elegant global trials have shown that anti-programmed cell death 1 or immune checkpoint inhibitors combined with chemotherapy significantly improve progression-free survival and overall survival in advanced non-squamous NSCLC compared with chemotherapy alone, regardless of programmed death-1 expression levels.^[8–10]^

To date, there have been no reported trials that directly compare pembrolizumab/carrelizumab monotherapy versus pembrolizumab/carrelizumab and chemotherapy in the first-line treatment setting of advanced/metastatic NSCLC. We performed a Bayesian network meta-analysis of randomized controlled trials (RCTs) to compare the efficacy and safety of pembrolizumab/carrelizumab versus pembrolizumab/carrelizumab and chemotherapy in previously treated patients with NSCLC.

## 2 Materials and methods

### 2.1 Data sources and search strategy

The following search terms would be used in PUBMED, Scopus, EMBASE, and Cochrane Library databases on July 20, 2021, as the search algorithm: (pembrolizumab) OR (carrelizumab) OR (programmed death-1) AND (non-small cell lung cancer) OR (NSCLC). Two searchers would independently draft and carry out the search strategy, and the third member would further complete it. No time limit was given to publication date. References within included articles were reviewed to include articles that were not included within our literature search. As this study was on the basis of published or registered studies, ethical approval and informed consent of patients were not required. The systematic review protocol had been registered on Open Science Framework registries. The registration number was 10.17605/OSF.IO/GYDV2. The systematic literature review was structured to adhere to PRISMA guidelines (Preferred Reporting Items for Systematic Reviews and Meta-analyses), which included requirements deemed essential for the transparent reporting of results. We would update our protocol for any changes in the entire research process if needed.

### 2.2 Eligibility criteria

All RCTs that reported the outcomes of pembrolizumab/carrelizumab with or without chemotherapy compared with those of pembrolizumab/carrelizumab alone for patients with NSCLC were considered eligible for inclusion in this meta-analysis. Patients with NSCLC were randomly assigned to receive either pembrolizumab/carrelizumab with or without chemotherapy or pembrolizumab/carrelizumab alone. Biomechanical studies, non-randomized cohort studies, in vitro studies, review articles, techniques, case reports, letters to the editor, and editorials were excluded.

### 2.3 Data extraction

Data extraction methods would follow the approach outlined by the Cochrane Handbook for Systematic Reviews of Interventions. Two independent authors extracted the following descriptive original information from the selected studies: study characteristics, such as author, year of publication, study design; Patient demographic information, such as number of patients, mean age, body mass index, and sex ratio. The primary outcomes of interest were overall survival, progression-free survival, objective response rate based on the Response Evaluation Criteria in Solid Tumors for complete and partial responses, and treatment-related adverse events including immune-related adverse events. Secondary outcomes included overall survival, progression-free survival, objective response rate, and treatment-related adverse events for the FDA-approved doses. If data were missing or could not be extracted directly, we would contact the appropriate author to ensure information integration. If necessary, we would forgo extracting incomplete data.

### 2.4 Statistical analysis

We used WinBUGS 1.4.3 software (MRC Biostatistics Unit, Cambridge, UK) and NetMetaXL (Canadian Agency for Drugs and Technologies in Health, Ottawa, Canada) to conduct a Bayesian network meta-analysis. Network meta-analysis combined data from several different randomized comparisons of different treatments to provide an internally consistent set of estimates, while respecting randomization in each trial. The network meta-analysis was performed within a generalized linear model framework with a link function that specified the relationship between the results and the model coefficients to be estimated. When the outcome was continuous, the likelihood was modeled as normal. When the outcome was the event rate, the likelihood was modeled as Poisson. The random effects model was used for this analysis. Estimation was performed in a Bayesian context using the non-information prior distribution of the parameters. The model was evaluated using the Deviation Information Criterion, a measure that combines model fit and complexity. The analysis was estimated using a Bayesian Markov Chain Monte Carlo model.

### 2.5 Quality assessment

The Cochrane Risk of Bias tool was used to assess the risk of bias in randomized clinical trials included by 2 independent reviewers. The quality of the randomized clinical trials was assessed using the following 7 items: random sequence generation, allocation concealment, blinding of participants and personnel, blinding of outcome assessment, incomplete outcome data, selective reporting, and other bias. The Kappa score, which measured agreement between two reviewers, was given as follows: fair, 0.40 to 0.59; good, 0.60 to 0.74; and excellent, 0.75 or more. Any disputes would be resolved by discussion with the third author to reach a final agreement.

## 3 Discussion

To date, there have been no reported trials that directly compare pembrolizumab/carrelizumab monotherapy versus pembrolizumab/carrelizumab and chemotherapy in the first-line treatment setting of advanced/metastatic NSCLC. We performed a Bayesian network meta-analysis of RCTs to compare the efficacy and safety of pembrolizumab/carrelizumab versus pembrolizumab/carrelizumab and chemotherapy in previously treated patients with NSCLC.

## Author contributions

**Conceptualization:** Xiangkun Qu.

**Data curation:** Xiaofei Zhang, Hui Qian.

**Formal analysis:** Xiaofei Zhang, Hui Qian.

**Funding acquisition:** Yalin Jiang.

**Investigation:** Xiaofei Zhang, Hui Qian.

**Methodology:** Xiangkun Qu.

**Project administration:** Yalin Jiang.

**Resources:** Yalin Jiang.

**Software:** Xiangkun Qu.

**Supervision:** Xiangkun Qu.

**Validation:** Hui Qian.

**Visualization:** Hui Qian.

**Writing – original draft:** Xiaofei Zhang.

**Writing – review & editing:** Yalin Jiang.
